# Critical Consciousness in Sport Scale (CCSS): development and initial psychometrics properties

**DOI:** 10.1186/s41155-025-00346-1

**Published:** 2025-04-25

**Authors:** Evandro Morais Peixoto, Martin Camiré, Amanda Rizzieri Romano

**Affiliations:** 1 São Francisco University (USF), 105 Waldemar César da Silveira St, Jardim Cura D’ars, Campinas, SP 13045 - 510 Brazil; 2 University of Ottawa, 125 University Private, Montpetit 345, Ottawa, ON K1 N 6 N5 Canada

**Keywords:** Critical reflection, Political efficacy, Critical action, Sport psychology, Psychometrics, Athletes

## Abstract

**Background:**

Critical consciousness refers to the ability to recognize and analyze the oppressive forces in society and to act against them. Researchers have emphasized that sport has the potential to help athletes develop their critical consciousness regarding the forces that shape society, as well as the systems of privilege and deprivation that influence access to sport.

**Objective:**

The present study describes the development of the critical consciousness in sport scale, offering initial validity evidence based on test content, internal structure, relations with other variables, and reliability.

**Methods:**

An initial set of 50 items was develop and reviewed by expert judges who assessed the clarity, practical relevance, and theoretical adequacy of the items using the content validity coefficient (CVC). To investigate the internal structure of the CCSS, factor retention methods such as parallel analysis and exploratory graph analysis were employed, followed by an exploratory factor analysis (EFA), along with an assessment of the internal consistency of the factors. Validity evidence based on relations with other variables was estimated using Pearson correlations. The sample was comprised of 263 Brazilian psychology and physical education students (mean age: 26.95 ± 9.69; 70.02% women).

**Results:**

Factor retention methods that included parallel analysis, exploratory graph analysis, and a categorical exploratory factor analysis demonstrated a three-dimensional structure comprised of 36 items, as theoretically hypothesized, with desirable internal consistency indices (*ω* = 0.868, 0.906, and 0.924, respectively). A brief version of the instrument is also presented, which adequately reproduced the psychometric properties of the initial version. Correlations with measurements of social justice and anti-racism efficacy suggested validity evidence based on relations with other variables.

**Conclusion:**

The results suggest that the instrument is an appropriate measure of critical consciousness in sport. It is recommended that future efforts focus on estimating further validity evidence and reliability of the CCSS in diverse samples of athletes from different competitive levels, sports coaches, fans, and other key figures within the sports context.

## Introduction

The sport environment is a context, when appropriately structured, which can foster physical, psychological, and social development, due to the experiences and social interactions that are made possible through sport (Bean et al., 2016; Holt et al., 2020, 2017). In relation to notions of development, the concept of social justice has been highlighted in the literature, in particular the potential of sport to help athletes develop their critical thinking skills towards the forces that shape society as well as the systems of privileges and deprivations influencing access to sport (Camiré et al., 2022; Darnell & Millington, 2019; Mac Intosh et al., 2020).

Social justice is a broad construct which can be understood as the fair distribution of resources, opportunities, and privileges in society (Rawls, 1971). Additionally, social justice is based on the tenet that all individuals in society should (a) have equal social, political, and economic rights,; (b) be open to diversity, equity, and inclusion; and (c) recognize social privileges and power relations (Camiré et al., 2022).

Examples of social injustices and movements to fight against these injustices are commonly observed in today’s society (Randall et al., 2023). These injustices and movements are also very much present in the context of sport. It has been widely publicized in the media and extensively documented in the scientific literature how instances of racism, homophobia, sexism, classism, and many other injustices are prevalent in sport. To fight against these injustices, some athletes use sport as a platform to make themselves heard (Turgeon et al., 2023). Examples were observed in relation to movements such as Black Lives Matter and Me Too where athletes from around the world confronted structural racism and police brutality against Black people while also fighting against sexual harassment and sexual aggression against women. However, athlete involvement in social justice movements does not occur by chance. It is highly dependent on their level of consciousness of the privileges and oppressions that exist inside/outside of sport (Turgeon et al., 2023). In this regard, the concept of critical consciousness is crucial to consider to appropriately address situations of oppression and discrimination in sport (e.g., Camiré et al., 2022; Gonzalez et al., 2020).

The term critical consciousness was proposed by Brazilian philosopher-educator Paulo Freire (1981) and refers to a person’s ability to recognize and analyze the oppressive forces that shape society and act against these forces. Thus, Freire (1981) used the term awareness or critical consciousness to refer to the combination of reflection and action on the world to transform it.

In the sports context, this implies that athletes and coaches should not only focus on technical skills but also understand the role of sport in society, promoting a more ethical and conscious practice (Camiré et al., 2012; Turgeon et al., 2023). This aligns with moral development, in which individuals learn to distinguish between right and wrong while considering the impact of their actions on the collective. Values in sport, such as fair play, respect, and solidarity, are fundamental to the moral development of athletes (Anjos, 2020). Critical consciousness can foster a deeper discussion about these values, helping athletes to understand not only the importance of respecting opponents and rules but also recognizing how these principles apply to life beyond the sports context. Thus, the role of social interaction and the environment in moral development is emphasized (Li et al., 2015).

According to Freire (1981), critical consciousness is composed of three components: critical reflection, political efficacy, and critical action. Critical reflection corresponds to consciousness of the structures of social inequality and the ways in which historical, cultural, and social processes perpetuate these inequalities over time. Also called social analysis, critical reflection refers to the ability to identify and analyze the social, political, and economic forces contributing to inequality. From this perspective, when oppressed people and groups engage in critical reflection, dominant narratives that hide or perpetuate oppression are challenged.

Political efficacy refers to an individual’s perceived capacity to promote social change. It stands as the personal belief about one’s ability to carry out social or political changes. Political efficacy is a crucial component of critical consciousness as it bridges an individual’s recognition of oppression and injustice (critical reflection) into a commitment to stand against these forces (critical action; Ibrahim et al., 2022).

Critical action corresponds to the sociopolitical actions individuals engage in to combat inequality, confront oppressive forces and structures, and challenge the unequal conditions that perpetuate these forces and structures. Critical actions for social justice can be individual or collective and include a wide range of approaches through which individuals challenge oppressive forces. In this regard, engagement in critical social actions is the ultimate goal of critical consciousness (Freire, 1981).

Studies examining the concept of critical consciousness have been conducted in different fields that include education (Seider & Graves, 2020; Tyler et al., 2019), social studies (Diemer & Rapa, 2016; Lee & Haskins, 2022), the arts (Ibrahim et al., 2022), and sport (Camiré et al., 2022; Newman et al., 2022; Turgeon et al., 2023). Systematic reviews suggested that the dimensions of critical consciousness were associated with a number of adaptive developmental outcomes, including career-related, civic, social-emotional, and academic outcomes (Heberle et al., 2020), and with indicators of well-being (mental, socioemotional, and physical health) (Ballard & Ozer, 2016; Castro et al., 2022). In another set of studies, measurement instruments assessing critical consciousness have been proposed (see Rapa et al., 2023). For example, Diemer and colleagues led studies on the construction, refinement, and validation of the *Critical Consciousness Scale* (CCS; Diemer et al., 2015, 2017). The initial version of the instrument consisted of 22 items, distributed between critical reflection (perceived inequality — 8 items; egalitarianism — 5 items) and critical action (sociopolitical participation — 9 items) factors. The psychometric properties of the CCS have been demonstrated in ethnically and economically diverse student samples. Diemer et al. (2022) proposed a short version of the instrument (short CCS), which is composed of 13 items distributed across critical reflection, critical motivation, and critical action dimensions. The short version of the instrument showed good psychometric properties when evaluated by factorial models and item response theory.

Other authors have also contributed to measurement efforts. Thomas et al. (2014) developed the *Critical Consciousness Inventory* (CCI), a unidimensional measure that evaluates perceptions of equity and justice in society as well as access to resources and educational opportunities for different social groups. In the educational context, the *Contemporary Critical Consciousness Measure* (CCCM; Shin et al., 2016) is an instrument composed of 19 items that assesses critical consciousness in relation to racism, classism, and heterosexism. There is also the CCCM-II (Shin et al., 2018), which expands the original CCCM to evaluate critical consciousness associated with sexism, cissexism (genderism/transphobia), and ableism.

It is emphasized that critical consciousness is not limited to the recognition of injustices but also motivates individuals to actively engage in the fight against them. Thus, it is characterized as a continuous process that goes beyond reflection, manifesting itself in practical actions aimed at promoting social justice. The literature indicates that critical consciousness can lead to positive outcomes, also influencing how individuals perceive their ability to promote social change (Bishop et al., 2023). In the context of the fight against racism, this phenomenon is even more evident. Study by Bañales et al. (2021) support this idea, showing that critical consciousness about injustices is positively associated with self-efficacy in anti-racist actions. Thus, when individuals develop a greater critical awareness of racial inequalities, they tend to feel more empowered to act effectively against racism. Although Freire (1981) did not specifically discuss anti-racist self-efficacy, his theory of conscientization is directly related to the idea that the perception of injustices and oppression can enhance the belief in one’s ability to transform these realities.

Moving forward, there is a need for an instrument evaluating expressions of critical consciousness in sport. Having an instrument of this nature can contribute to assessing the level of critical consciousness of athletes as well as those who operate in this context (e.g., coaches, parents, managers, psychologists, physiotherapists, among other sports science professionals). In efforts to assess critical consciousness while being mindful of the psychological, environmental, and social variables relevant to the context of sport (Bishop et al., 2023), the present research aims to develop the Critical Consciousness in Sport Scale (CCSS) by providing initial validity evidence based on content, internal structure, relations with external variables (social justice and anti-racism efficacy), and accuracy. To this end, it is hypothesized that (a) the set of items developed will adequately cover the measurement of the critical consciousness dimensions; (b) the instrument must recover the structure composed of three dimensions (critical reflection, political efficacy, and critical action), in line with the theoretical perspective (Freire, 1981); (c) the factors will present good reliability indicators; and (d) the factors will present relations with external variables consistent with the theoretical perspectives, that is, the factors that compose critical consciousness must be positively related to social justice and anti-racism efficacy; according to these theoretical frameworks, individuals’ awareness of social injustices and oppression should translate into actions and attitudes that promote social justice and enhance anti-racist efficacy. Critical consciousness, as articulated by Paulo Freire (1981), encompasses not only the recognition of existing inequalities but also an active commitment to combating those inequalities. Therefore, it is anticipated that the factors comprising critical consciousness will be positively associated with behaviors and beliefs that advocate for social justice and actively challenge racism.

## Method

### Participants

The convenience sample consisted of 263 university students aged between 18 and 67 years (*M* = 26.95 ± 9.69), of which 70.72% declared themselves cisgender women, 27% cisgender men, 1.52% preferred not to answer, and 0.76% declared themselves to be non-binary. Of the participants, 71.86% considered themselves white, 18.63% brown, 7.22% Black, 1.52% Asian, and 0.76% Indigenous. Regarding marital status, 79.09% declared themselves single, 19.01% married, 1.14% divorced, and 0.76% widowed. Concerning education level, 86.93% were enrolled in an undergraduate course, 11.41% in *stricto *Sensu postgraduate studies (masters/doctorate), and 1.66% in *lato *sensu postgraduate courses and represented psychology (74.52%), physical education (24.33%), and sports science (1.15%). It is worth mentioning that 43.72% of participants reported practicing sport, 19.1% performed professionally in the context of sport, and all of them have practiced at least one sport in their lifetime.

### Instruments

#### Characterization questionnaire

Built specifically to meet the objectives of the study, the instrument aims to collect sociodemographic information, such as age, gender, educational level, and sports practice.

#### Anti‑Racism Efficacy Scale (A-RES, Eschmann, 2023)

A-RES is a self-report instrument composed of four items that aims to evaluate indicators of anti-racism self-efficacy: (a) the self-perceived ability to face racism (e.g., *You consider yourself well qualified to participate in movements related to racism*) and (b) the degree of confidence that the respondent has in promoting changes when combating racism (e.g., *People like you can influence the way racism affects others*). The A-RES items are answered using a 4-point Likert scale (ranging between 0 = *strongly disagree* and 4 = *strongly agree*). The psychometric properties of the A-RES demonstrated the suitability of a single-factor structure and good accuracy indicators (Eschmann, 2023). The adaptation and validation of the Brazilian version of the A-RES corroborate the adequacy of the internal structure, as well as an adequate internal consistency value, and McDonald’s omega was 0.68 (Romano et al., 2024). The McDonald’s omega coefficient estimated in our sample was 0.703.

#### Social Justice Scale (SJS, Torres-Harding et al., 2012)

The SJS is a self-report instrument composed of 24 items that assesses four components of social justice (social justice-related behaviors): attitudes towards social justice (e.g., “I believe that it is important to make sure that all individuals and groups have a chance to speak and be heard, especially those from traditionally ignored or marginalized groups”), social justice perceived behavioral control (e.g., “I am confident that I can have a positive impact on others’ lives”), social justice subjective norms (e.g., “Other people around me are engaged in activities that address social injustices”), and social justice behavioral intentions (e.g., “In the future, I will do my best to ensure that all individuals and groups have a chance to speak and be heard”). The SJS items are answered using a 7-point Likert scale (ranging from 1 = *strongly disagree* to 7 = *strongly agree*). Studies based on the internal structure of the SJS indicated adequacy of the four-factor structure and good levels of reliability (Torres-Harding et al., 2012). Adaptation study and validation of the Brazilian version corroborate the adequacy of the internal structure and reliability. The McDonald’s omega coefficient estimated for the respective factors in our sample was 0.978, 0.925, 0.898, and 0.945, similar to the original study (attitudes *α* = 0.95, perceived behavioral control *α* = 0.84, subjective norms *α* = 0.82, and intentions *α* = 0.88) (Torres-Harding et al., 2012).

#### Critical consciousness in sport scale (CCSS)

Built and structured in the present study, the CCSS is an instrument composed of items that assess the three aspects that compose critical consciousness: critical reflection, political efficacy, and critical action. To answer the questionnaire, individuals must read each item and indicate the degree to which they agree with each statement, expressed using a 5-point Likert scale (1 = *strongly disagree* and 5 = *strongly agree*).

### Procedures

#### Construction of the CCSS and content validity evidence

To construct the CCSS, the criteria specified by Pasquali (2010) were met, stating that the items should express the content through behaviors and present simplicity, clarity, relevance, and precision in relation to the factors. Regarding the set of items, we sought to construct items that represented different expressions of the target construct and, therefore, covered a considerable range of critical consciousness dimensions. Thus, it was expected to compose an instrument with the potential to assess people with different levels of critical reflection, political efficacy, and critical action. To prepare the items, we consulted instruments developed to assess critical consciousness in other contexts (e.g., education; Diemer et al., 2017; Thomas et al., 2014), as well as sport-specific instruments that touched on dimensions of critical consciousness (Shin et al., 2016, 2018). Initially, 50 items were developed in the format of statements, reflecting the three dimensions of critical consciousness (Rapa et al., 2023) and covering the four central themes relevant to sport: racism (e.g., “Black people are victims of racism in sport”), gender inequality (e.g., “I can contribute to fostering gender equality in sport”), sexism (e.g., “I support on social media the LGBTQ community in sport”), and socioeconomic inequality (e.g., “I participate in activities that promote social equality in sport”). It is worth mentioning that due to the nature of the construct evaluated (i.e., critical consciousness), the items were developed by taking into account the neutralization proposal, which aims to reduce the evaluative load of the contents presented in the items (Bäckström, 2007). Negative items were also developed for the instrument, which makes it possible to model and control response biases with the influence of acquiescence through the modeling of the random intercept factor (see Aichholzer, 2014; Valentini et al., 2018). These procedures have been successfully used in the literature (Bäckström, 2007; Costa & Hauck Filho, 2017; Peixoto et al., 2019) to develop instruments less influenced by social desirability and acquiescence biases.

The items were initially evaluated for adequacy by three expert researchers/PhDs in the areas of psychology, pedagogy, and sociology of sport who have extensive knowledge in Freirean theorization. To estimate content validity, five independent judges (i.e., PhDs in psychology with knowledge in psychometrics, sport psychology, and familiarity with the concept of critical consciousness) evaluated the clarity, practical relevance, and theoretical adequacy of the items. The calculation of the content validity coefficients (CVC; Hernández-Nieto, 2002) indicated the clarity of the language used in the items, with CVC values for clarity between 0.89 and 1. CVC values for practical relevance were between 0.92 and 0.98, demonstrating the importance of the items composing the CCSS. Finally, CVC values for theoretical adequacy were between 0.98 and 1, demonstrating the suitability of the items in terms of their association with the dimensions that make up critical consciousness. The judges also made qualitative remarks to improve the contents of the items, suggesting the use of appropriate and coherent terms for the characteristics of the target population. All remarks were accepted and incorporated into the items.

To further refine the instrument and gather additional evidence of content validity, two separate focus-group discussions were conducted with representatives of the target population for whom the CCSS is intended. The first group consisted of professionals, including three sport psychology practitioners and two sport science professionals. The second group included students: six undergraduate psychology students and three students from sport science programs. Each group reviewed the items independently, focusing on interpretability, clarity, and appropriateness to ensure the content was accessible and meaningful to their respective contexts. Feedback was collected on the wording, cultural alignment, and potential interpretability challenges. Overall, participants indicated that the items were appropriate and well-constructed. As a result, no significant modifications to the content of the items were necessary.

### Ethical aspects

The project was submitted to the Research Ethics Committee of the first author’s university, and upon approval, all the materials were uploaded to a Google Forms online platform. The Google Forms link was shared on social networks, in professional contact networks, and in classrooms. Prior to answering the questions, participants completed the free and informed consent form (TCLE, for its acronym in Portuguese). By agreeing to participate in the research and stating that they were 18 years of age or older, participants were provided access to the materials, presented in the following order: sociodemographic questionnaire, CCSS, SJS, and A-RES. All questionnaires/scales took approximately 20 min to complete. The datasets used and/or analyzed during the current study are available from the corresponding author on reasonable request.

### Data analysis

Initially, evidence was investigated based on the internal structure of the CCSS. To this end, methods were used to assess factorial retention, such as parallel analysis (Timmerman & Lorenzo-Seva, 2011) and exploratory graph analysis (Golino & Epskamp, 2017). The factorial solution was estimated using exploratory factor analysis (EFA) with an estimation method appropriate to the ordinal measurement level: unweighted least squares (ULS) and Promim rotation method (Lorenzo-Seva, 1999), based on polychoric correlation matrices (Holgado–Tello, 2010). To establish the factor model, the interpretability of the set of items grouped into the factors was considered, and the representativeness of the items in the factor/value of factor loadings needed to be greater than 0.32 (Costello & Osborne, 2019). Additionally, we sought to maintain at least three items for each major theme evaluated by the dimensions that compose the CCSS theoretical model (critical reflection, political efficacy, and critical action) and prioritize the maintenance of negative items in the respective factors.

The reliability indicators of the respective factors that make up the instrument were also verified. To this end, McDonald’s Omega measure of internal consistency was used. As indicated by specialized literature, indexes equal to or greater than 0.70 were considered as reference values (Tabachnick et al., 2019). These analyses were carried out with the support of the statistical software Factor v. 10.8.04 (Lorenzo-Seva & Ferrando, 2013).

With the first version of the CCSS and the estimation of the first validity evidence based on the internal structure, the objective was to develop a short version of the instrument. The search for an abbreviated version of the instrument, capable of preserving the psychometric characteristics of the long version, reflects the practical challenges faced by researchers and professional sports psychologists (Horvarth & Röthlin, 2018), since the use of extensive instruments can be tiring and detrimental to the quality of the data obtained (Alcaraz et al., 2020). Additionally, it may be limited due to the restrictions of the sporting environment itself; therefore, instrument simplification is necessary.

The goal was to (a) maintain two items for each major theme under evaluation (racism, homophobia, classism, sexism) in each CCSS factor, (b) retain items with higher factor loadings in the initial version (Costello & Osborne, 2005), and (c) keep at least one negative item in the factors that contained such items. Additionally, the content of the items was checked to ensure proper evaluation of the construct’s extension and, thus, the maintenance of the validity and reliability indicators observed in the original version (Marsh et al., 2020). To evaluate the internal structure of the brief version, confirmatory factor analysis (CFA; Marsh et al., 2020) was implemented, carried out using the Mplus software (Muthén & Muthén, 2017). The CFA was conducted with an estimation method suitable for ordinal variables: weighted least square (WLSMV), based on the polychoric correlation matrix, since the variables are ordinal (Izquierdo et al., 2014). The following fit indices were used: chi-square (*χ*^2^), degrees of freedom (df), root-mean-square error of approximation (RMSEA), comparative fit index (CFI), and Tucker–Lewis Index (TLI). To interpret the results, the following parameter adequacy values were used: *χ*^2^/df < 5, *RMSEA* ≤ 0.08, CFI and TLI ≥ 0.95, and factor loadings greater than or equal to 0.3 (Muthén & Muthén, 2017; Tabachnick & Fidell, 2019).

To estimate validity evidence based on the relations with other variables, an analysis of the association between the scores estimated in the initial and brief versions of the CCSS and the measures of social justice and anti-racism self-efficacy was carried out. To this end, Pearson correlations were employed using the Jamovi software (2020). Levels of *p* < 0.05 were considered significance indicators, and the magnitudes of the correlations were interpreted based on the classification proposed by Cohen (1988), with − 0.09 to 0.09 described as null, 0.10 to 0.29 small, 0.30 to 0.49 medium, and 0.50 to 1.0 large. Lastly, group comparisons based on participants’ gender (male and female) were conducted for the dimensions of the CCSS using Cohen’s *t*-test to identify potential differences. The selection of a parametric correlation technique was based on the assumption of a normal distribution for the variables, with skewness and kurtosis values ranging between − 2 and 2.

## Results

### Internal structure — initial version

**Fig. 1:**
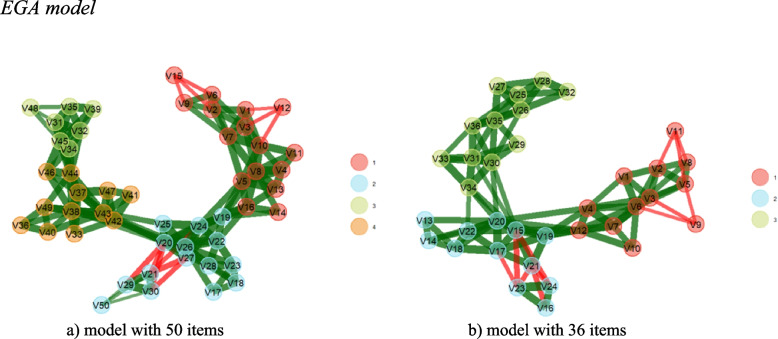
EGA model. **a** model with 50 items. **b** model with 36 items

Initially, the adequacy indicators of the factorial matrix for carrying out the EFA were checked, which indicated adequacy to the data, *KMO* = 0.91685, Bartlett’s *χ*^2^ = (990) 2385.1, and *p* < 0.00001. To retain factors, parallel analysis and EGA were used, which indicated the relevance of extracting up to four factors. It is noted, through PA, that only the first four factors estimated through real data presented percentages of explained variances (35.39; 14.76; 6.96 and 4.60, respectively) higher than the values allocated in the 95 th percentile among the data random (5.39; 5.02; 4.78 and 4.54, respectively). The 50-item factorial structure model proposed by the EGA model can be seen in Fig. 1.

From Fig. 1a, the first grouping of items (red nodes) corresponds to the items developed to evaluate the critical reflection factor, and the second grouping (blue nodes) corresponds to political efficacy items. Groups 3 (green nodes) and 4 (orange nodes) correspond to a division of critical action items. However, these items had high correlations between them (represented in the figure by green edges), which was corroborated by the estimation of the exploratory factor model, as the items largely presented cross-factorial loading between these factors.

Therefore, the EFA was carried out forcing the solution to retain three factors, which presented a good fit to the data. In other words, only four items were excluded because they did not present a factorial loading above 0.32 in their original factors. Subsequently, the criteria established for the present research were applied, with the aim of establishing a scale with a parsimonious number of items, that is, maintaining the items with the greatest representation in the construct (factor loadings), retaining three items for each major theme evaluated (racism, homophobia, classism and sexism) in the dimensions that compose the theoretical model (critical reflection, political efficacy, and critical action), and prioritizing the maintenance of negative items in the respective factors.

Factor retention methods (parallel analysis and EGA) were estimated again, which showed adequacy of the internal structure composed of three factors. Since only the first four factors estimated using real data presented percentages of explained variances (35.95; 15.36; 7.80 and 5.31, respectively) higher than the values allocated to the 95 th percentile among the random data (6.64; 6.09; 4.78 and 5.74, respectively), the model proposed by EGA is presented in Fig. 1b, where it can be seen that the first grouping of items (red nodes) corresponds to the 12 items referring to the critical reflection factor, the second grouping (blue nodes) to the 12 items referring to political efficacy, and the third grouping (green nodes) to the 12 items developed for the assessment of critical action.

In Table 1, the CCSS exploratory factor model is presented. Moreover, the factor loadings of the items, the correlations between the factors, and the reliability estimates are described.

**Table 1:** CCSS factor model

	EFA initial version			CFA brief version		
**Items**	**CR**	**PE**	**CA**	**CR**	**PE**	**CA**
PE01	−.036	− 0**.643**	.048	0.791		
PE02	− 0.248	**0.899**	.019	0.773		
PE03	− 0.146	**0.908**	−.013	0.789		
PE04	0.357	**0.591**	− 0.211			
PE05	−.059	**0.763**	−.013	0.750		
PE06	.014	**0.729**	.096	0.957		
PE07	0.259	**0.552**	− 0.112			
PE08	− 0.139	**0.596**	− 0.115	0.688		
PE09	−.003	− **0.404**	− 0.101	− 0.475		
PE10	− 0.115	**0.625**	0.191	0.705		
PE11	.001	− **0.392**	.067			
PE12	0.218	**0.558**	.014			
CR01	**0.727**	− 0.106	−.061		0.610	
CR02	**0.782**	−.038	−.081			
CR03	**0.690**	0.156	0.119			
CR04	− **0.565**	0.113	− 0.154			
CR05	**0.677**	.065	−.012		0.876	
CR06	**0.812**	−.093	−.064		0.831	
CR07	**0.680**	0.296	−.024		0.893	
CR08	**0.805**	.061	.085		0.909	
CR09	**0.814**	.064	.008		0.853	
CR10	**0.910**	− 0.133	.001		0.848	
CR11	− **0.523**	0.174	− 0.193			
CR12	− **0.561**	0.130	−.082		− 0.552	
CA01	.032	− 0.118	− **0.721**			0.902
CA02	.038	− 0.112	**0.844**			0.927
CA03	0.109	− 0.191	**0.791**			0.828
CA04	−.092	−.059	**0.839**			0.839
CA05	.068	.094	**0.694**			
CA06	.034	0.187	**0.715**			0.756
CA07	−.061	0.188	**0.775**			
CA08	.021	−.005	**0.770**			0.827
CA09	.042	0.196	**0.579**			
CA10	0.131	− 0.168	**0.655**			
CA11	−.009	− 0.140	**0.935**			0.884
CA12	−.001	−.021	**0.815**			0.807
Factor	CR	PE	CA	CR	PE	CA
CR	-			-		
PE	0.553	-		0.511	-	
CA	0.207	0.484	-	0.198	0.456	-
ɷ	0.868	0.906	0.924	0.854	0.911	0.896

*EFA* Exploratory Factor Analysis; *CFA* Confirmatory Factor Analysis; *CCSS* Factors; *CR* critical reflection; *PE* political efficacy; *CA* critical action; ɷ McDonald's Omega

As evidenced in Table 1, there was no cross-loading of items with a value greater than or equal to 0.30. The correlation matrix between factors demonstrated relations that varied between 0.198 and 0.553. According to PA, the three-factor model was able to explain 59.11% of the variance in the data. Finally, as shown, the variables presented good reliability indicators for all factors (*ɷ* > 0.70).

### Confirmatory factor analysis

In order to assess the contribution of the negative items from the CCSS, we estimated a confirmatory model of its initial version, both with and without the inclusion of a random intercept (Maydeu-Olivares & Steenkamp, 2018). This approach relaxes the assumption of a common intercept for all respondents, allowing the intercept to vary from respondent to respondent. By accounting for this variability, the model can more accurately reflect individual differences in response tendencies, which may otherwise distort the measurement of latent constructs. This flexibility is particularly useful in mitigating the effects of response biases, such as acquiescence. In this case, all loadings of the additional factor are fixed at 1, and the estimated score can be interpreted as a form of “response bias score,” reflecting the degree to which respondents tend to endorse the item, regardless of its content (Martins et al., 2023).

The model without acquiescence control demonstrated a good fit: *χ*^2^ (df) = 1413.309 (591), *p* < 0.001, *RMSEA* = 0.079 (90% *CI* [0.074–0.084]), *CFI* = 0.930, and *TLI* = 0.925. The positive factor loadings for the CR factor ranged from 0.642 (item 9) to 0.919 (item 7), while the negative items had loadings of − 0.435 (item 15) and 0.489 (item 12). For the EP factor, positive loadings ranged from 0.608 (item 17) to 0.898 (item 26), while negative loadings were − 0.671 (item 21), − 0.785 (item 29), and − 0.784 (item 30). The AC factor loadings ranged from 0.747 (item 41) to 0.895 (item 32). Correlations between factors ranged from 0.563 (CR and PE) to 0.270 (CR and CA).

The confirmatory factor model with a random intercept showed slight improvements in fit indices: *χ*^2^ (df) = 1254.424 (590), *p* < 0.001, *RMSEA* = 0.071 (90% CI [0.066–0.077]), *CFI* = 0.944, and *TLI* = 0.940. However, there was a reduction in positive factor loadings and an increase in negative factor loadings. For instance, the CR factor loadings ranged from 0.586 (item 9) to 0.871 (item 7), with negative items loading at − 0.570 (item 15) and − 0.621 (item 12). For the EP factor, positive loadings ranged from 0.550 (item 17) to 0.852 (item 26), while negative loadings were − 0.741 (item 21), − 0.826 (item 29), and − 0.825 (item 30). The CA factor loadings ranged from 0.695 (item 41) to 0.855 (item 32). Lastly, the acquiescence factor loadings were 0.266, indicating that about 0.07% of the item’s response variance could be attributed to acquiescence. The correlations between factor remained similar to the model without acquiescence control, ranging from 0.539 (CR and PE) to 0.202 (CR and CA). These findings highlight the importance of retaining negative items in the instrument due to their potential for controlling response bias and its effects on the estimation of internal structure.

After estimating the first evidence based on the internal structure of the CCSS, we sought to propose a brief version of the instrument. To this end, the items that presented the highest factor loadings for each of the dimensions were used as item selection criteria. Furthermore, the internal structure and reliability of the short version of the CCSS were evaluated. The results are presented in Table 1. With regard to model adjustment, the parameters indicated results that were considered good: *χ*^2^ (df) = 540.547(249) × *p* < 0.001, *RMSEA* = 0.072 (90% *CI* [0.064–0.081]), *CFI* = 0.964, and *TLI* = 0.961. The items produced factor loadings close to those found in the long version of the instrument (≥ − 0.475) and also presented significant correlations between the factors, with factor loadings varying between 0.198 and 0.511. In general, the results obtained suggest that even with an abbreviated instrument, there was no loss in terms of access to the scope of the psychological construct (i.e., critical consciousness) assessed in the long version.

### Relations with external variables

Regarding the objective of searching for validity evidence based on the relations with external variables, a correlation analysis was carried out with the Pearson index (*r*). As can be seen in Table 2, the factors referring to CCSS were positively and significantly related to the factors that make up social justice, with the exception of (a) critical reflection with social justice perceived behavioral control and (b) critical action with social justice attitudes and Anti-Racism Efficacy. The magnitudes of the relations varied between weak and strong. Regarding the correlation estimates of the brief version, the magnitudes observed in the initial version were maintained. Finally, the correlations between the respective factors of the initial and brief versions are highlighted, and the results indicate a very high correlation (*r* > 0.969).

**Table 2:** Correlation between critical consciousness in sport, social justice, and anti-racism self-efficacy variables

		**Critical consciousness ** **in sport**			**Social justice**				**Anti-racism self-efficacy**
**Factors**	**x̄ (±)**	**CR**	**PE**	**CA**	**SJA**	**SJBC**	**SJSN**	**SJBI**	**ARE**
CR	4.27 (0.63)	0.980**							
PE	4.07 (0.76)	0.460*	0.979**						
CA	2.26 (0.95)	0.224*	0.472*	0.969**					
SJA	6.43 (1.07)	0.414*	0.432*	.092	-				
SJBC	5.36 (1.31)	.094	0.395*	0.225*	0.455*	-			
SJSN	4.82 (1.09)	0.191*	0.378*	0.300*	0.366*	0.482*	-		
SJBI	5.82 (1.04)	0.352*	0.474*	0.284*	0.634*	0.609*	0.553*	-	
ARE	2.72 (0.47)	0.314*	0.538*	0.298*	0.281*	0.526*	0.312*	0.433*	-

The correlations presented on the lower diagonal correspond to the estimate between the initial version and the external variables, while those presented on the upper diagonal refer to the brief version. On the diagonal, highlighted in bold, the correlations between the factors of the initial version and brief version are presented

**p>0.05; x̄(±)= Mean(Standard-Deviation); CCSS Factors: CR = Critical Reflection, PE = Political Efficacy, CA = Critical Action; Social Justice Factors: SJA = Social Justice Attitudes, SJBC = Social Justice Perceived Behavioral Control, SJSN = Social Justice Subjective Norms, SJBI = Social Justice Behavioral Intentions; ARE = Anti-racism Efficacy

Table 2 also presents the mean scores obtained on the response scales. Regarding critical consciousness, it is noteworthy that the CR and PE factors showed high mean scores, close to the maximum possible score (5). Regarding social justice, the SJA factor had the highest mean score, while the SJSN factor had the lowest mean score.

Table 3 presents the mean differences based on the participants’ sex (female and male). As can be observed, for the three factors that make up the CCSS (CR, PE, and CA), the observed mean differences were not statistically significant, with *p*-values exceeding the significance threshold of 0.05. Furthermore, the Cohen’s d values indicate that the effect size is small in all cases, with CR showing slightly larger effect, but still considered small in statistical terms. Therefore, despite the observed mean differences between the groups, they are not statistically significant, and the effect size suggests that these differences, even if they exist, have limited practical relevance.

**Table 3:** Independent samples t-test based on sex

Factors CCSS	Group	*N*	*M*	SD	*p*	MD	SE	Cohen’s *d*
CR	F	186	4.28	0.60	0.563	0.672	1.16	0.088
	M	71	4.22	0.69				
PE	F	186	4.09	0.75	0.915	0.150	1.40	0.016
	M	71	4.07	0.77				
CA	F	186	2.22	0.93	0.159	− 2.454	1.74	− 0.215
	M	71	2.43	0.98				

Note. *p > .05; *d < .30; Group: F = female, M = male; CCSS Factors: CR = Critical Reflection, PE = Political Efficacy, CA = Critical Action; M = Mean, SD = Standard-deviation, MD = Mean Difference, SE = Standard Error of the Difference

## Discussion

The objective of the present study was to develop the CCSS, as well as to gather validity evidence and reliability in a sample composed of Brazilian university students. To this end, different statistical methods were used in accordance with confirmed validity evidence, namely: content, internal structure, relations with external variables, internal consistency, and operationalization of a short version of the instrument. In general, the data obtained demonstrate the suitability of the instrument through a structure composed of three factors related to each other (critical reflection, political efficacy, and critical action), which corroborates the theoretical proposal (Freire, 1981). Good accuracy rates were also observed for all dimensions of the instrument. Furthermore, as hypothesized, the factors that compose critical consciousness demonstrated positive relation with social justice and anti-racism self-efficacy.

Among the sources of validity evidence that can be used to evaluate instruments, there is validity evidence based on the test content (AERA et al., 2014). Such evidence explores the level to which the content of the items corresponds to the construct being assessed by the instrument. Based on the results obtained in the evaluation process by expert judges, who considered cultural, linguistic, and subjective aspects of the target construct, it was found that the content of the items was efficient based on the CVC (≥ 0.80). These preliminary results therefore indicate the first evidence of content-based validity of the CCSS.

Regarding the internal structure, the EFA results indicated the adequacy of the model and structure of the instrument’s items, reproducing a model of three correlated factors, in accordance with the theoretical proposal (Freire, 1981). These results also corroborate those found in studies carried out with available instruments applied in different areas (Diemer et al., 2017, 2022), thus demonstrating the measure’s ability to recover the theoretical structure that supported the item development. It is worth highlighting that the correlations between the scale’s factors corroborated the inferences of the measure’s coherence. A high correlation was observed between critical reflection and political efficacy factors, confirming the theoretical hypothesis that the perceived individual capacity to confront injustices and promote social changes in sports is associated with the understanding of the oppressive forces underlying expressions of inequality. In the same direction, a moderate positive correlation between political efficacy and critical action was verified, suggesting that the use of critical actions is associated with this self-perceived individual capacity to promote the necessary changes. Finally, a positive correlation of low magnitude was observed between critical reflection and critical action factors, which indicates that the simple recognition and awareness of oppressive forces in the context of sports do not guarantee engagement in actions and movements to combat these injustices (Torres-Harding et al., 2012). As, in agreement with Freire (1981, 1993), it is precisely the combination of high levels in these factors that comprises critical consciousness/awareness, as well as the potential of oppressed and marginalized people to reflect and confront unjust political and social conditions (Diemer et al., 2017, 2022; Ginwright & James, 2002).

Still on the internal structure, the strategy of constructing negative items for the CCSS appears to be appropriate, as well as the efforts to retain them across different versions of the instrument, given the scale’s potential to control response biases such as acquiescence. As highlighted in the literature, the inclusion of a random intercept model can help to mitigate the impact of these biases on the internal structure of the instruments, thus improving the accuracy of latent variable estimation. It is important to note that failing to account for this control could lead to distorted estimates of the instrument’s internal structure and, consequently, inaccurate respondent characteristic estimates (Martins et al., 2024; Maydeu-Olivares & Steenkamp, 2018). The findings of the present study align with both simulation studies (Martins et al., 2023) and empirical research (Campos et al., 2024; Padilha et al., in press), highlighting the importance of including negative items and controlling for acquiescence, even in unbalanced scales (i.e., those where the number of positive and negative items are not equivalent).

Regarding the reliability indicators of the factorial structure, the reliability indices obtained in this study presented solid results. McDonald’s omega demonstrated satisfactory performance in all dimensions of the instrument. This highlights the accuracy in measuring critical consciousness in sport and the instrument’s ability to assess the constructs with a reduced estimation error associated with the participants’ scores (Tabachnick & Fidell, 2019). The set of these results allows us to infer the first validity evidence based on the internal structure and reliability of the CCSS in a sample of Brazilian adults (AERA et al., 2014). The results observed in this research indicate an important contribution to the field of critical consciousness measurement development, given that measures incorporating all three dimensions of critical consciousness are just emerging (Rapa & Godfrey, 2023). Finally, the assessment of critical consciousness related to specific themes (racism, sexism, homophobia, and classism) and contexts (sports) also represents current advancements in the field and refers to the stage named by Rapa et al. (2023) as “Scale Expansion and (Re)Specification.”

Furthermore, the study aimed to propose a brief version of the CCSS. With regard to the psychometric properties of this version, the results observed, through CFA, suggested validity evidence based on the internal structure and internal consistency, replicating the factorial structure of the initial version and maintaining high levels of internal consistency. It is worth highlighting that the correlations between the factors, when estimated by the brief version, maintained the same magnitude pattern observed in the initial version, as well as the fact that the correlations between the estimated scores between the versions were equal to or greater than 0.97. This demonstrates, therefore, that the two alternatives are efficient and measure critical consciousness in sport in a similar way, that is, without loss of content coverage (Diemer et al., 2022).

The strategy of maintaining the most representative items, keeping at least two items for the evaluation of the themes emphasized in the construction of the instrument in each factor (racism, classism, sexism and homophobia), and maintaining at least one negative item in the CR and PE factors contributed to reproducing the quality of the initial version of the CCSS. Therefore, the present study dedicated efforts to produce an option that offers good psychometric qualities and facilitates application in different sporting and researching environments (Horvarth & Röthlin, 2018).

Finally, regarding the validity evidence based on the relations with external variables, it is noted that the observed relations are in line with the theoretical rationale that underlies the instruments. Regarding the critical reflection factor, the highest correlations stand out with the factors that presented components of analysis and understanding of power relations in society, namely: *social justice attitudes* (which correspond to the level of acceptance of social justice ideals, values, and beliefs that one should act in a fair and equal manner in society), *social justice behavioral intentions* (behavioral intentions to engage in social actions or activities related to social justice in the near future), and *anti-racism efficacy* (competence and self-perceived abilities to confront racism).

The political efficacy factor, in line with the theoretical model (Freire, 1981), showed higher correlations with indicators of perceived competence to face social injustice, such as the *anti-racism efficacy* scale, suggesting that the perceived capacity for confronting classism, homophobia, sexism, and racism in sport is associated with the perception of confronting racism in society (Eschmann et al., 2023). It is worth highlighting that although the level of magnitude of correlation is high, a relevant part of the variance of the variables is not shared, which is appropriate, as the items in the CCSS political efficacy factor assess self-efficacy in confronting expressions of injustice that go beyond racism, as well as being restricted to the expressions of these injustices in the context of sports. The same reasoning can be used to interpret the moderate associations with *social justice attitudes*, *social justice perceived behavioral control*, and *social justice behavioral intentions* factors, which also present components of the self-perceived capacity to face expressions of social injustice, and intention to engage in behaviors and movements of this nature in the near future. Also, the *social justice subjective norms* factor encompasses the perception of support provided by the environment in which the individual is inserted to confront social injustices.

Finally, positive, significant, but low magnitude correlations were observed between the critical action factor and external variables. Such results are consistent with the understanding that in bivariate relations between the recognition of social expressions of injustice/racism, the perception of capacity to face these injustices, social and environmental support, and the intention to engage in behavior to confront injustices in the near future differentiate themselves from real engagement in social justice behaviors and movements in sports (Turgeon et al., 2023).

The set of results observed from the study of the relations with external variables allows inferring the adequacy of efforts to create a measure of critical consciousness specific to the context of sports, as well as inferring the first validity evidence based on the relations with CCSS external variable (AERA et al., 2014). It is also worth highlighting the maintenance of the correlation patterns estimated with the initial and brief versions of the CCSS with the external variables. Such results corroborate the suitability of the brief version of the instrument with regard to maintaining external validity evidence (Horvath Röthlin, 2018).

The means and standard deviations estimated for the CCSS indicators, along with the comparison of scores between men and women, indicate that respondents from both groups found it easier to endorse items related to CR and PE compared to those related to CA. These findings align with the theoretical framework, which suggests that recognizing social injustices and oppressive forces within one’s context does not necessarily translate into engagement in social change actions. This highlights the importance of creating opportunities for critical actions as a means to foster political efficacy and critical reflection, contributing to the holistic development of critical consciousness (Diemer et al., 2021).

From the point of view of practical implications, the development of the CCSS has potential for the assessment of sports science professionals, sports coaches, students, athletes, fans, among other characters in the sports scene, regarding individual differences in relation to the components of consciousness criticism in sport. The operationalization of the construct from the scale will allow evaluating individuals’ scores on these factors and also estimating the relations of these constructs with other individual, social, and contextual variables that can act as antecedents or results of critical consciousness in sport (Camiré et al., 2022; Gonzalez et al., 2020), as well as other variables related to social justice behavior in this context (Turgeon et al., 2023; Schinke, 2018).

It is important to emphasize that this study and the proposed new scale aim to fill a significant gap in the literature on critical consciousness in sports (Turgeon et al., 2023). The proposed scale measures the three components of critical consciousness: critical action, critical reflection, and political efficacy. By addressing these components, the scale empowers athletes and professionals to recognize and challenge the injustices and inequalities present in the sporting environment and in society at large. This is crucial for fostering a culture of respect and inclusion in sports (Gonzalez et al., 2020).

Furthermore, critical consciousness contributes to the development of ethical and moral skills (Li et al., 2015). By understanding the implications of their actions and decisions, athletes and coaches can make more informed choices both on and off the field. This not only enhances the dynamics of sports but also contributes to the development of more responsible and socially engaged citizens (Camiré et al., 2022). The new scale allows sports professionals to address issues such as racism, gender inequality, and other forms of discrimination in a more informed and effective manner. Finally, by providing a robust tool for measuring these components, the scale can serve as a foundation for future research, advancing knowledge in the field and promoting more conscious and ethical practices in sports. Therefore, the importance of our proposal extends beyond academia, directly impacting sports practice and society.

### Limitations and future directions

From the results obtained in this research, it can be inferred that the established objectives were achieved. However, limitations of the study can be indicated, such as the characteristics of a non-probabilistic sample, composed mainly of university students, cisgender women, from the Southeast Region of Brazil. Another limitation concerns the use of a cross-sectional research design to evaluate associations with external variables, which prevents the inference of causality between the variables. To address these limitations, it is suggested that studies be carried out with longitudinal designs to increase understanding of how these variables interact over time, as well as estimating the relations with external criteria.

Additionally, it is suggested that new efforts be made to estimate validity evidence and reliability of the CCSS and diverse samples. In this sense, studies of this nature can be reproduced with samples of athletes from different competitive levels, sports coaches, fans, among other characters from the sports scene. Finally, it is considered important to develop validity evidence studies based on the response process (Hubley, 2021), especially with participants who represent ethnic, social, and gender diversity. This will allow the identification of the cognitive trajectory, as well as experiences retrieved by respondents to answer the items, enabling the understanding of how these experiences are equivalent, or not, between groups. Also, the accumulation of this evidence will allow greater knowledge about how the content of the items affects the respondents.

It is also important to highlight the advantages and limitations of administering the survey through Google Forms. The online application facilitates access from any device with Internet connectivity, allowing a larger number of participants to complete the survey at their own convenience. Furthermore, responses are collected in real time, enabling immediate access to data for analysis, which can expedite the research process. However, this approach may lead to less careful responses, as participants might rush through the survey without adequately reflecting on the questions. Additionally, the administration of online surveys limits the opportunity for real-time clarification of questions or concerns, which can impact the quality of the data collected. In light of these considerations, it is suggested that future studies also administer the instrument in a pencil-and-paper format to assess the invariance between the two response formats.

Furthermore, it is suggested that future studies develop an extra short version of the CCSS. To this end, it is recommended to use item response theory to estimate the item parameters (e.g., difficulty and discrimination) and with this a better understanding of how the items evaluate the extent of the construct. It is also recommended that future studies invest in the development of new items to capture the dimensions of critical consciousness for the assessment of other themes not evaluated in the present research, such as ableism, taking into account the expressions of social injustices experienced by paralympic athletes (see Howe, 2019).

## Conclusions

This study made it possible to confirm the potential of the CCSS to assess critical consciousness in sport in the Brazilian population by demonstrating the stability of the instrument as well as the internal structure, reliability, and relationship with external variables. The findings of this first study provide support for the psychometric properties of the initial and brief versions of the CCSS and stimulate the continuity of research into the construct of critical consciousness using these instrument in future studies in order to assess the antecedents and consequences of the three dimensions of critical consciousness in sport and the replication of results found in this study, which will contribute to the accumulation of validity evidence to support the interpretation of the CCSS scores.

## Data Availability

The datasets used and/or analyzed during the current study are available from the corresponding author on reasonable request.

## Abbreviations

A-RES: Anti‑Racism Efficacy Scale
CCCM: Contemporary Critical Consciousness Measure
CCI: Critical Consciousness Inventory
CCS: Critical Consciousness Scale
CCSS: Critical Consciousness in Sport Scale
CFA: Confirmatory factor analysis
CFI: Comparative fit index
CI: Confidence interval
CVC: Content validity coefficient
EFA: Exploratory factor analysis
EGA: Exploratory graph analysis
KMO: Kaiser–Meyer–Olkin
LC: Language clarity
DF: Degrees of freedom
RF: Rigid persistence
RMSEA: Root-mean-square error of approximation
SJS: Social Justice Scale
TLI: Tucker–Lewis index
ULS: Unweighted least squares
WLSMV: Weighted least squares mean and variances adjusted
